# A multi-institutional randomized phase III study comparing minimally invasive distal pancreatectomy versus open distal pancreatectomy for pancreatic cancer; Japan Clinical Oncology Group study JCOG2202 (LAPAN study)

**DOI:** 10.1186/s12885-024-11957-9

**Published:** 2024-02-19

**Authors:** Naoki Ikenaga, Tadayoshi Hashimoto, Junki Mizusawa, Ryo Kitabayashi, Yusuke Sano, Haruhiko Fukuda, Kohei Nakata, Kazuto Shibuya, Yuji Kitahata, Minoru Takada, Keiko Kamei, Hiroshi Kurahara, Daisuke Ban, Shogo Kobayashi, Hiroaki Nagano, Hajime Imamura, Michiaki Unno, Amane Takahashi, Shintaro Yagi, Hiroshi Wada, Hirofumi Shirakawa, Naoto Yamamoto, Seiko Hirono, Naoto Gotohda, Etsuro Hatano, Masafumi Nakamura, Makoto Ueno, on behalf of the Hepatobiliary and Pancreatic Oncology Group in Japan Clinical Oncology Group

**Affiliations:** 1 Department of Surgery and Oncology, Graduate School of Medical Sciences, Kyushu University, 3-1-1 Maidashi, 812-8582 Fukuoka, Japan; 2 Japan Clinical Oncology Group Data Center/Operations Office, National Cancer Center Hospital, Tokyo, Japan; 3 Department of Gastroenterology and Gastrointestinal Oncology, National Cancer Center Hospital East, Kashiwa, Japan; 4 Translational Research Support Office, National Cancer Center Hospital East, Kashiwa, Japan; 5 Department of Surgery and Science, Faculty of Medicine, Academic Assembly, University of Toyama, Toyama, Japan; 6 Second Department of Surgery, Wakayama Medical University, Wakayama, Japan; 7 Department of Surgery, Teine Keijinkai Hospital, Hokkaido, Japan; 8 Department of Surgery, Kindai University Faculty of Medicine, Osakasayama, Japan; 9 Department of Digestive Surgery, Kagoshima University, Kagoshima, Japan; 10 Department of Hepatobiliary and Pancreatic Surgery, National Cancer Center Hospital, Tokyo, Japan; 11 Department of Gastroenterological Surgery, Graduate School of Medicine, Osaka University, Suita, Japan; 12 Department of Gastroenterological, Breast and Endocrine Surgery, Yamaguchi University Graduate School of Medicine, Yamaguchi, Japan; 13 Department of Surgery, Nagasaki University Graduate School of Biomedical Sciences, Nagasaki, Japan; 14 Department of Surgery, Tohoku University Graduate School of Medicine, Sendai, Japan; 15 Department of Gastroenterological Surgery, Saitama Cancer Center, Saitama, Japan; 16 Department of Hepato-Biliary-Pancreatic Surgery and Transplantation, Kanazawa University, Ishikawa, Japan; 17 Department of Gastroenterological Surgery, Osaka International Cancer Institute, Osaka, Japan; 18 Department of HepatoBiliary-Pancreatic Surgery, Tochigi Cancer Center, Tochigi, Japan; 19 Department of Gastrointestinal Surgery, Kanagawa Cancer Center, Kanagawa, Japan; 20 Division of Hepato-Biliary-Pancreatic Surgery, Department of Gastroenterological Surgery, Hyogo Medical University, Hyogo, Japan; 21 Department of Hepatobiliary and Pancreatic Surgery, National Cancer Center Hospital East, Chiba, Japan; 22 Department of Surgery, Graduate School of Medicine, Kyoto University, Kyoto, Japan; 23 Department of Gastroenterology, Kanagawa Cancer Center, Kanagawa, Japan

**Keywords:** Clinical trial, Laparoscopy, Minimally invasive surgical procedures, Pancreatectomy, Pancreatic neoplasm

## Abstract

**Background:**

Minimally invasive distal pancreatectomy (MIDP), including laparoscopic and robotic distal pancreatectomy, has gained widespread acceptance over the last decade owing to its favorable short-term outcomes. However, evidence regarding its oncologic safety is insufficient. In March 2023, a randomized phase III study was launched in Japan to confirm the non-inferiority of overall survival in patients with resectable pancreatic cancer undergoing MIDP compared with that of patients undergoing open distal pancreatectomy (ODP).

**Methods:**

This is a multi-institutional, randomized, phase III study. A total of 370 patients will be enrolled from 40 institutions within 4 years. The primary endpoint of this study is overall survival, and the secondary endpoints include relapse-free survival, proportion of patients undergoing radical resection, proportion of patients undergoing complete laparoscopic surgery, incidence of adverse surgical events, and length of postoperative hospital stay. Only a credentialed surgeon is eligible to perform both ODP and MIDP. All ODP and MIDP procedures will undergo centralized review using intraoperative photographs. The non-inferiority of MIDP to ODP in terms of overall survival will be statistically analyzed. Only if non-inferiority is confirmed will the analysis assess the superiority of MIDP over ODP.

**Discussion:**

If our study demonstrates the non-inferiority of MIDP in terms of overall survival, it would validate its short-term advantages and establish its long-term clinical efficacy.

**Trial registration:**

This trial is registered with the Japan Registry of Clinical Trials as jRCT 1,031,220,705 [https://jrct.niph.go.jp/en-latest-detail/jRCT1031220705].

## Background

Minimally invasive distal pancreatectomy (MIDP), including laparoscopic and robotic distal pancreatectomies, has gained widespread acceptance over the last decade with the improvements in operative procedures and development of surgical instruments. Multiple meta-analyses of retrospective studies have shown that, compared with open distal pancreatectomy (ODP), MIDP results in reduced estimated blood loss and shorter hospital stays [1–3], which has been confirmed by a well-designed multicenter randomized controlled study [4]. Although the safety of MIDP for short-term outcomes has been well documented, evidence regarding its long-term oncologic outcomes is limited. This gap is primarily due to the absence of randomized controlled trials assessing long-term outcomes despite a few retrospective studies that have indicated comparable oncologic outcomes between MIDP and ODP [5, 6].

A meta-analysis of 21 studies involving 11,246 patients who underwent distal pancreatectomy for pancreatic cancer revealed comparable proportions of microscopic radical resection (R0 resection) and survival after MIDP and ODP [5]. Notably, MIDP was more often performed on smaller and less aggressive tumors, suggesting that treatment selection bias strongly affected the results. A propensity score-matched cohort study comprising 340 patients who underwent each of these procedures also demonstrated comparable overall survival for patients with pancreatic cancer treated with either MIDP or ODP [6]. In this analysis, the patient’s physiological and oncological characteristics were matched between the treatment groups to minimize treatment selection bias. However, lymphovascular and perineural tumor invasion were observed more frequently in MIDP than in ODP, and the number of retrieved lymph nodes was lower in MIDP despite matching. Moreover, the surgical techniques and pathological assessments were not standardized, and several data were missing or underreported. Owing to the retrospective nature of the study, the results of this study need to be interpreted with caution. Evidence Map of Pancreatic Surgery provided by the International Study Group of Pancreatic Surgery [7] has shown five RCTs and seven ongoing trials on the topic of MIDP versus ODP (as of January 2024). Four of the reported RCTs evaluated short-term outcomes or the quality of life in MIDP, and oncologic outcomes were analyzed in only one RCT, with the R0 resection rate as the primary endpoint [8]. The primary endpoints of the ongoing trials are recurrence-free survival in one, 2-year survival rate in one, and short-term outcomes in the other five trials. Details on the progress of these ongoing trials have not been disclosed.

Because of the lack of sufficient evidence regarding the oncologic safety of MIDP, its implementation in pancreatic cancer treatment remains controversial. A worldwide survey on minimally invasive pancreatic surgery demonstrated that 18% of surgeons across 50 countries considered pancreatic cancer a contraindication for MIDP [9]. International evidence-based guidelines endorsed by eight major surgical societies worldwide state that MIDP for pancreatic cancer appears to be an oncologically equivalent technique, particularly in the hands of experienced surgeons; however, additional randomized trials are recommended to strengthen the level of evidence [10]. The 2019 Clinical Practice Guidelines for Pancreatic Cancer from the Japan Pancreas Society acknowledge the potential benefits of MIDP for treating pancreatic cancer; however, the recommendation strength is weakly graded owing to limited available evidence [11]. Consequently, the implementation of MIDP for malignant diseases remains low at 17% in Japan [12], depriving most patients with pancreatic cancer of the benefits of minimally invasive surgery.

Given this background, we have launched a randomized controlled trial to compare the overall survival of patients with resectable pancreatic cancer treated with MIDP versus ODP. This study aims to confirm whether MIDP is a viable treatment option for pancreatic cancer.

## Methods

### Objectives

This study aims to confirm the non-inferiority of the overall survival of patients undergoing MIDP with regional lymph node dissection to ODP for resectable pancreatic cancer.

### Study setting

**Fig. 1:**
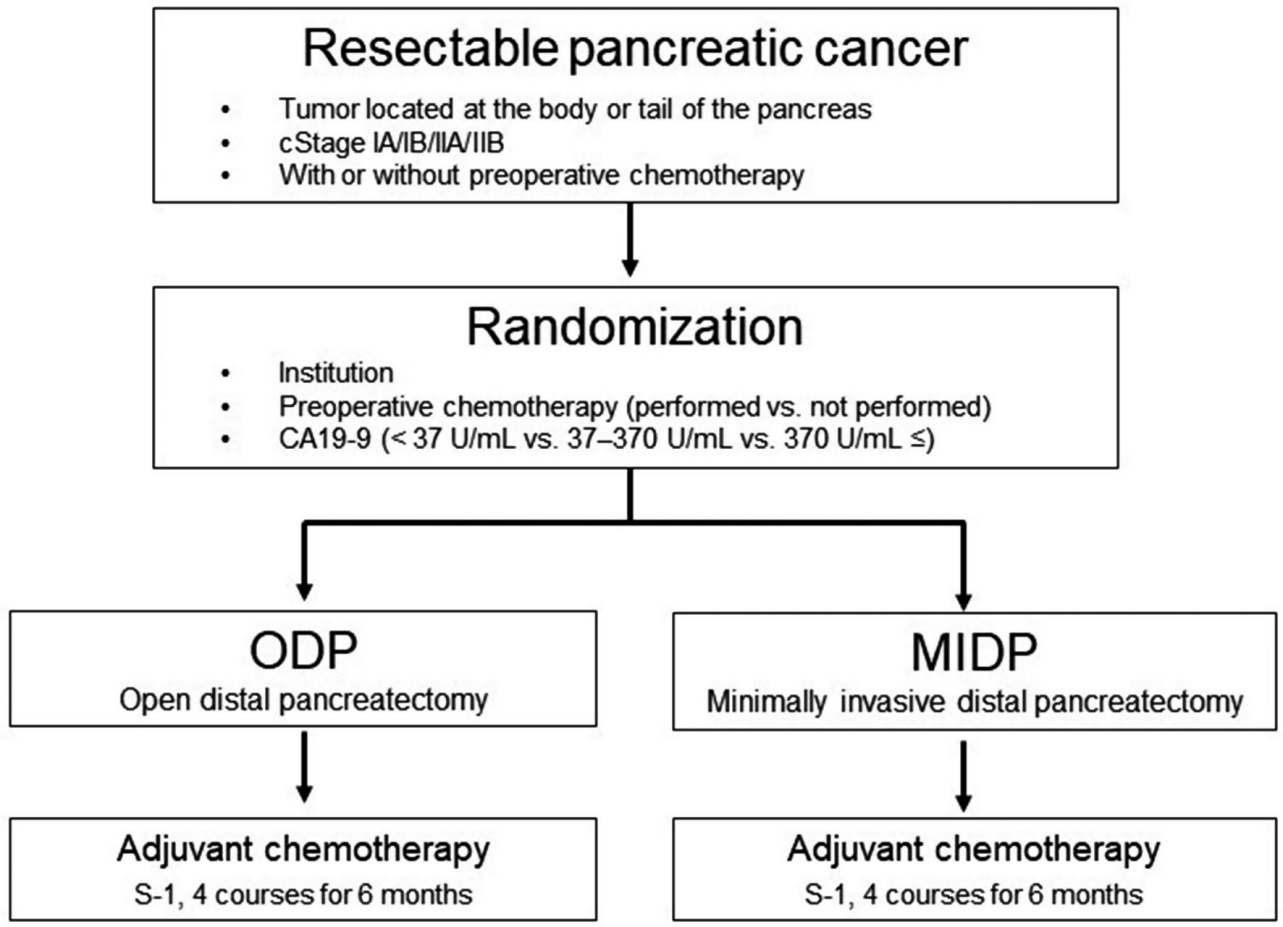
Schematics of this study

This is a multi-institutional, randomized, phase III study being conducted at 40 specialized centers. A schematic of the study is illustrated in Fig. 1.

### Institutional review boards

The Japan Clinical Oncology Group (JCOG) Protocol Review Committee approved the study protocol in December 2022. The National Cancer Center Hospital Certified Review Board approved the study protocol in January 2023, and patient enrollment began in March 2023. This trial is registered in the Japan Registry of Clinical Trials under the number jRCTs1031220705 [https://jrct.niph.go.jp/en-latest-detail/jRCT1031220705].

### Endpoints

The primary endpoint of this study is overall survival, measured within the per-protocol population, including all patients who undergo the assigned surgical approach with curative intent. Patients who change their chemotherapy regimen will not be excluded from the per-protocol population. Overall survival is defined as the time from randomization to death from any cause, with censoring at the last recorded date when the patient is still alive.

The secondary endpoints are relapse-free survival, proportion of patients undergoing R0 resection, operation time, estimated blood loss, maximum incision length, proportion of patients undergoing complete laparoscopic surgery, incidence of adverse surgical events, perioperative mortality, and length of postoperative hospital stay. Relapse-free survival is defined as the time from randomization to relapse or death from any cause with censoring at the last recorded date when the patient is still alive without any evidence of relapse. The proportion of patients undergoing R0 resection is defined as those who successfully undergo R0 resection among those who undergo the assigned surgical approach with curative intent. Maximum incision length refers to the measurement of the longest of all skin incisions. The proportion of patients who complete laparoscopic surgery is defined as those in the MIDP arm who undergo laparoscopic or robotic surgery without requiring conversion to open surgery among those who undergo the assigned surgical approach with curative intent. Adverse events are assessed according to the Common Terminology Criteria for Adverse Events (CTCAE) version 5.0 [13] and the Clavien–Dindo classification for surgical complications [14].

### Eligibility criteria

Patients are required to fulfill all of the following criteria:

1. Pancreatic tumors diagnosed using contrast-enhanced abdominal computed tomography (CT) meeting either one of the following conditions:

- Histologically proven invasive ductal carcinoma (adenocarcinoma or adenosquamous carcinoma)^*^.
- Cytologically proven Class IV or Class V.

* In case of the following condition of a) or b), pancreatic tumor radiologically compatible with invasive ductal carcinoma is eligible without a pathological diagnosis.

- No preoperative chemotherapy.
- Endoscopic ultrasound-guided tissue acquisition or endoscopic retrograde cholangiopancreatography cannot be safely performed.

2) Tumor located in the body or tail of the pancreas.
3) Resectable pancreatic cancer determined by contrast-enhanced CT. Patients treated with preoperative chemotherapy are required to be diagnosed with resectable tumors before and after preoperative chemotherapy.
4) Maximum length of the tumor ≤ 8 cm.
5) Curative resection is possible using distal pancreatectomy and regional lymph node dissection.
6) In the case of the preceding diagnostic laparoscopy, peritoneal cytology is negative.
7) No invasion to the portal vein or other organs, except for the adrenal gland or spleen.
8) Patients aged between 18 and 85 years.
9) Performance status (Eastern Cooperative Oncology Group) of 0 or 1.
10) No prior radiation therapy against pancreatic cancer.
11) No history of upper abdominal surgery, except for laparoscopic cholecystectomy.
12) Sufficient organ functions:
  - Neutrophil ≥ 1,200/mm^3^.
  - Hemoglobin ≥ 9.0 g/dL.
  - Platelet ≥ 75,000/mm^3^.
  - Total bilirubin ≤ 2.5 mg/dL.
  - Aspartate aminotransferase ≤ 150 IU/L.
  - Alanine transaminase ≤ 150 IU/L.
  - Creatinine ≤ 1.5 mg/dL.

13) Provision of written informed consent by the patient.

### Exclusion criteria

Patients with any of the following criteria are excluded:

1. Synchronous or metachronous (within 5 years) malignancies, except for cancers with a 5-year relative survival rate of ≥ 95%, such as carcinoma in situ, intramucosal tumor, or early-stage cancers.
2. Infectious disease that requires systemic treatment.
3. Body temperature of ≥ 38.0 °C.
4. Pregnant women, those within 28 days of the post-parturition phase, lactating mothers, or men with partners expecting conception.
5. Severe psychiatric conditions affecting daily life.
6. Receiving continuous systemic corticosteroid or immunosuppressive treatment.
7. Severe comorbidities (heart failure, renal failure, liver failure, hemorrhagic peptic ulcer, intestinal obstruction, and poorly controlled hypertension).
8. History of unstable angina pectoris within 3 weeks or myocardial infarction within 6 months before registration.
9. Diagnosis of interstitial pneumonia, pulmonary fibrosis, or severe emphysema through chest radiography.
10. Contraindication to iodide because of allergy, renal failure, or bronchial asthma.

### Randomization

After confirming the eligibility criteria, registration is performed using a web-based system at the JCOG Data Center. The patients are randomized (1:1) to the ODP or MIDP arm by minimization method, incorporating a random component to balance the arms based on institution, history of preoperative chemotherapy (performed vs. not performed), and the carbohydrate antigen 19 − 9 (CA19-9) level (< 37 U/mL vs. 37–370 U/mL vs. 370 U/mL ≤).

### Surgical approach

All procedures, except the surgical approach, were performed in a similar manner. ODP or MIDP is performed in the respective arms after intraperitoneal assessment confirms that the tumor is resectable. Intraoperative rapid cytology diagnosis is mandatory, and the operation is terminated when the cytology is positive. Preoperative or intraoperative staging laparoscopy is performed in both arms. The extent of nodal dissection was determined based on tumor location. The regions of the pancreas and lymph node stations are defined according to the General Rules for the Study of Pancreatic Cancer 7th Edition from the Japan Pancreas Society [15]. Lymph node stations 10, 11d, 11p, and 18 are dissected from tumors located in the tail of the pancreas. Lymph node stations 8a, 9, 10, 11d, 11p, 14p, 14d, and 18 are dissected from the tumors located in the body or both the body and tail of the pancreas. The transection line and method of pancreatic resection are not specified at each institution; however, the same approach applies to both MIDP and ODP at each institution. Examination of frozen sections at the cut end of the pancreas is not mandatory. Combined splenectomy is performed in all patients. The surrounding pancreas tissues, including the renal fascia, are dissected to achieve > 1 mm clear resection margins for the tumor.

In the MIDP arm, a mini-laparotomy incision of > 8 cm is not allowed. Surgical assistance by robots for laparoscopic procedures is allowed; thus, laparoscopic and robotic distal pancreatectomies are included in the MIDP arm. If intraoperative findings reveal any of the following conditions, MIDP is converted to open surgery:

1. Combined resection of the portal vein, common hepatic artery, or celiac artery is required because of tumor invasion.
2. Combined resection of the colon or combined resection of the stomach with gastroenteric reconstruction is required because of tumor invasion. A partial gastrectomy that does not require gastroenteric reconstruction is allowed.

### Quality control of the surgery

Institutions participating in this study are restricted to board-certified institutions of the Japanese Society of Hepato–Biliary–Pancreatic Surgery (JSHBPS). Only surgeons credentialed by the study chair can be responsible for ODP and MIDP, and only board-certified experts or instructor surgeons qualified by the JSHBPS can be credentialed surgeons in both arms. The JSHBPS board certification system requires expert surgeons to have experience with 50 or more high-level hepatobiliary–pancreatic (HBP) surgeries, as defined by the JSHBPS as the primary operator. Furthermore, they must pass a video review that evaluates their surgical skills in performing high-level HBP surgeries [16]. In the MIDP arm, credentialed surgeons also need to have experience with 10 or more MIDPs and be certified or have an equivalent certification from the Japan Society for Endoscopic Surgery [17]. For robotic surgery, experience with five or more robotic distal pancreatectomies is required. All ODP and MIDP procedures are centrally reviewed using intraoperative photographs of the dissected field, and the maximum length of the skin incision is centrally reviewed using postoperative photographs.

### Adjuvant chemotherapy

Patients in both arms receive four cycles of oral S-1 twice daily for 4 weeks, followed by a 2-week rest period. Three dose levels of S-1 are determined according to the body surface area (BSA) as follows: BSA < 1.25 m^2^, 40 mg; BSA 1.25–1.50 m^2^, 50 mg; BSA > 1.50 m^2^, 60 mg, twice a day. The treatment is to be continued for up to 24 weeks or until the detection of relapse; appearance of unacceptable toxicities, such as grade 4 non-hematological toxicities; or patient refusal.

### Patient follow-up

Patients are followed up for a minimum of 4 years after completion of patient enrollment. Enhanced CT of the upper abdomen and pelvis, chest CT, and serum tumor marker levels, including carcinoembryonic antigen and CA19-9, are evaluated every 3 months for 3 years and every 6 months for the remaining 2 years. Relapse is diagnosed based on CT findings. Physical and laboratory examinations are performed at least once every 6 weeks during S-1 treatment. Subsequently, these examinations are performed every 3 months for 3 years and every 6 months for the remaining 2 years after enrollment. Toxicities are evaluated according to the CTCAE version 5.0 [13].

### Study design and statistical analysis

This randomized controlled trial is designed to demonstrate that MIDP is not inferior to ODP in terms of overall survival. Some endpoints have been adopted to evaluate the safety of MIDP and the lower invasiveness of MIDP over ODP, but all these endpoints are considered exploratory. Therefore, as long as the non-inferiority of MIDP is confirmed, MIDP will be considered a standard treatment option for resectable pancreatic cancer. According to the Schoenfeld and Richter method [18], the required sample size is 314 patients (244 deaths), with 157 patients per arm. We anticipate 4 years of follow-up after 4 years of enrollment, ensuring at least 70% power with a one-sided alpha of 5% and a non-inferiority margin of 10% in terms of 3-year survival. This corresponds to a hazard ratio of 1.32, using the hypothesis of an expected 3-year overall survival of 46% in each arm. Considering that 14% of the enrolled patients do not undergo pancreatectomy because of the detection of unresectable factors on intraoperative assessment [19], the required sample size in the present study is 365 patients. The total sample size is set to 370 patients, accounting for those that may be lost to follow-up. Patients randomized to the MIDP arm and converted to the ODP arm are included from the MIDP population for efficacy and safety analyses. The superiority of MIDP to ODP in terms of overall survival is also tested when the non-inferiority of MIDP to ODP is statistically proven.

### Interim analysis and monitoring

We plan to conduct interim analyses twice, accounting for multiplicity using the Lan–DeMets α-spending function with the O’Brien and Fleming type [20]. The first interim analysis will be performed after half of the planned number of patients has been enrolled. The second interim analysis will be performed after enrolling the entire planned number of patients and completing all protocol treatments. The Data and Safety Monitoring Committee of the JCOG will review the interim analysis reports independently from the group investigators and statisticians and judge whether the present trial should be terminated. If the superiority of the MIDP arm is demonstrated through the stratified log-rank test with a p-value lower than the adjusted alpha level, the study will be terminated. In-house monitoring will be conducted every 6 months by the JCOG Data Center to evaluate and improve the progress and quality of the study.

## Discussion

The JCOG 2202 (a multi-institutional randomized phase III study comparing MIDP versus ODP for pancreatic cancer) is the first randomized trial to evaluate the long-term outcomes of MIDP for resectable pancreatic cancer. A recent European study consortium reported the non-inferiority of MIDP to ODP in terms of the R0 resection rate [8]. Nonetheless, it is imperative to emphasize that the trial included 9% of non-malignant cases determined by postoperative pathological examination, and the per-protocol analysis did not establish non-inferiority. Moreover, the clinical utility of MIDP over ODP in terms of long-term prognostic outcomes remains unclear. JCOG2202 sets overall survival as primary endpoint in patients with pancreatic cancer who underwent distal pancreatectomy as primary endpoint, excluding non-malignant cases for the analysis. If our study demonstrates the non-inferiority of MIDP in terms of overall survival, it would validate its short-term advantages and establish its long-term clinical efficacy.

### Participating institutions (from north to south)

Hokkaido University Hospital, Sapporo Medical University, Teine-Keijinkai Hospital, Tohoku University Hospital, Fukushima Medical University, Tochigi Cancer Center, Saitama Prefectural Cancer Center, National Cancer Center Hospital East, Chiba University Hospital, National Cancer Center Hospital, Nihon University Itabashi Hospital, Kyorin University Hospital, Tokyo Medical University Hospital, Center Hospital of the National Center for Global Health and Medicine, Tokyo Women’s Medical University Hospital, Cancer Institute Hospital of Japanese Foundation for Cancer Research, Teikyo University Hospital, Tokai University Hospital, Kanagawa Cancer Center, Kitasato University Hospital, Niigata Cancer Center Hospital, Toyama University Hospital, Kanazawa University Hospital, Ishikawa Prefectural Central Hospital, Aichi Cancer Center Hospital, Kyoto University Hospital, Osaka University Hospital, Kindai University Hospital, Osaka International Cancer Institute, National Hospital Organization Osaka National Hospital, Kansai Medical University Hospital, Kobe University Hospital, The Hospital of Hyogo College of Medicine, Wakayama Medical University Hospital, Yamaguchi University Hospital, National Hospital Organization Shikoku Cancer Center, National Kyushu Cancer Center, Kyushu University Hospital, Nagasaki University Hospital, Kagoshima University Hospital.

## Data Availability

No datasets were generated or analysed during the current study.

## Abbreviations

CT: Computed tomography
CTCAE: Common Terminology Criteria for Adverse Events
HBP: Hepatobiliary–pancreatic
JCOG: Japan Clinical Oncology Group
JSHBPS: Japanese Society of Hepato–Biliary–Pancreatic Surgery
MIDP: Minimally invasive distal pancreatectomy
ODP: Open distal pancreatectomy
